# Symptomatic Aortic Paravalvular Leak: Percutaneous Treatment with Amplatzer Vascular Plug III Device as an Alternative to Surgery

**DOI:** 10.21470/1678-9741-2017-0103

**Published:** 2018

**Authors:** Gabriel E. Pérez Baztarrica, Gastón Heredia, Juan Arellano, Juan Fernández, Rafael Porcile

**Affiliations:** 1 Department of Cardiology and Physiology, University Hospital of the Faculty of Medicine of the Universidad Abierta Interamericana, Buenos Aires, Argentina.

**Keywords:** Heart Valve Prosthesis, Heart Failure, Anemia, Hemolytic, Cardiac Catheterization/Methods, Prosthesis Failure/*Adverse Effects

## Abstract

A significant prosthetic paravalvular leak is an uncommon and severe postsurgical complication correlated to the occurrence of congestive heart failure and hemolytic anemia. Percutaneous treatment has become an attractive and effective proposal to relieve symptoms and reduce complications in patients whose high rate of morbidity/mortality precludes a new surgery.

This is the case of an 81-year-old patient with a history of biological aortic valve replacement seeking medical help due to heart failure and hemolytic anemia, with a prosthetic paravalvular regurgitation jet and high surgical mortality according to EuroSCORE II.

**Table:** 

Abbreviations, acronyms & symbols	
AV	= Aortic valve
LDH	= Lactate dehydrogenase
NYHA	= New York Heart Association
OA	= Occlusive Amplatzer
PVL	= Paravalvular leak

## INTRODUCTION

A significant prosthetic paravalvular leak (PVL) is an uncommon and severe postsurgical complication correlated to the occurrence of congestive heart failure and hemolytic anemia. Percutaneous treatment has become an attractive and effective proposal to relieve symptoms and reduce complications in patients whose high rate of morbidity/mortality precludes a new surgery^[1]^.

This is the case of an 81-year-old patient with a history of biological aortic valve replacement seeking medical help due to heart failure and hemolytic anemia, with an 8.5 mm prosthetic paravalvular regurgitation jet and high surgical mortality according to EuroSCORE II^[2]^.

## CASE REPORT

An 81-year-old male patient was admitted due to New York Heart Association (NYHA) Functional Class III-IV refractory congestive heart failure despite optimal medical treatment (angiotensin-converting enzyme inhibitors, beta-blockers and angiotensin II receptors antagonists, under high doses of oral furosemide). The patient had a history of arterial hypertension, chronic renal failure and pulmonary obstructive chronic disease. In 2001, he underwent myocardial revascularization surgery, as well as aortic valve replacement with a biological prosthesis. In 2003, the patient experienced an ischemic stroke.

Upon admission, lab tests showed impaired kidney function (urea 88 g/dL and creatinine 2.1 mg/dL), anemia (Hb 7 g/dL), lactate dehydrogenase (LDH) (1100 mg/dL), and grade 3 to predominantly indirect bilirubin (based on patient's history and complementary tests, hemolytic anemia was assumed).

**Fig. 1:**
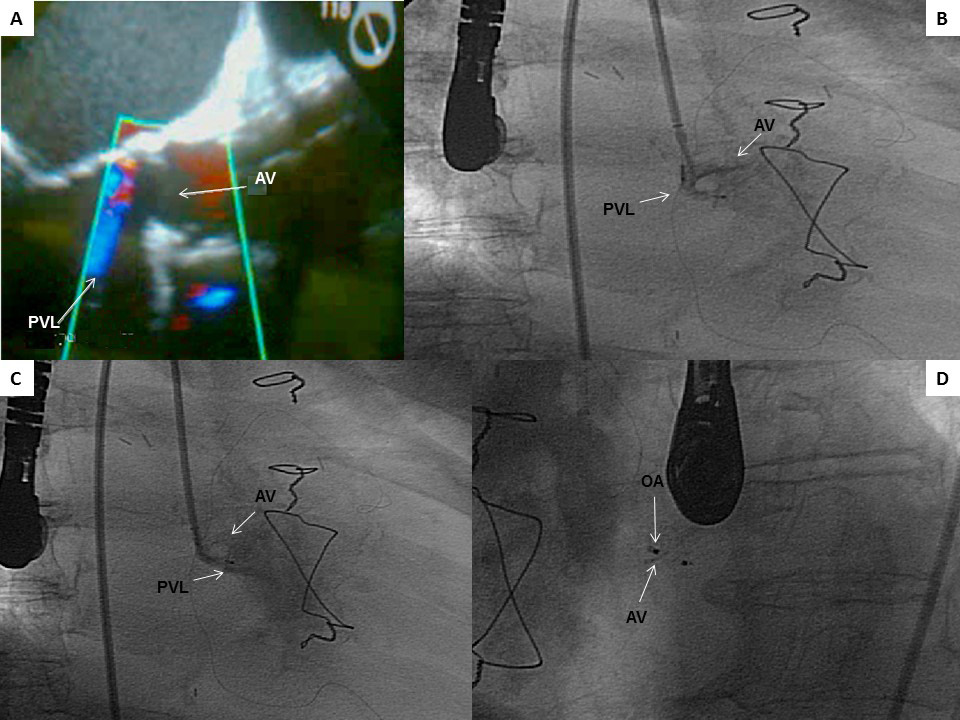
A) View of the aortic paravalvular regurgitant jet in a transesophageal echocardiogram. B and C) Picture showing the multipurpose catheter in an angiography to identify the leak. D) Amplatzer positioned to check location prior to occlusive device implantation. AV=aortic valve; OA=occlusive Amplatzer; PVL=paravalvular leak

A multiplanar transthoracic and transesophageal echocardiography was performed, which showed severely damaged ventricular function and mechanical prosthetic valve in a bileaflet normal function aortic position with a PVL, leading to severe regurgitant jet (8.5 mm wide and going into the middle third of the left ventricle). The regurgitant area was 43 mm^2^, with no images compatible with vegetations (Figure 1A).

The EuroSCORE II^[2]^ was estimated in order to anticipate surgical mortality and assess surgical risk. Mortality rate was estimated at 53.41%. Considering this high mortality rate, percutaneous closure of the PVL was planned.

In the hemodynamic laboratory, the patient was hemodynamically instable, under mechanical ventilation and sedoanalgesia by an anesthesiologist. Before the procedure, 10,000 IU of sodium heparin were administered together with infective endocarditis prophylaxis, a procedure led by fluoroscopy and transesophageal echocardiogram.

The PVL was closed retrogradely with a right femoral artery puncture, using an 8 French introducer. First, a multipurpose catheter angiography was conducted to see the PVL (Figures 1B and C). A wire-guided multipurpose catheter was placed towards the aortic valve plane. A Terumo hydrophilic guidewire was inserted in the catheter in order to go through the PVL, and then exchange was performed via a high-support guidewire (Amplatz). Afterwards, the Amplatzer Vascular Plug III (St. Jude Medical, Plymouth, MN, USA) release system was placed, and once on the dehiscence, the transesophageal echocardiography showed the end of the leak regurgitation jet. Then, the occlusion device was released (Figure 1D), after lack of interference with the mechanical prosthesis was ensured.

The procedure was successful, and immediately after the implantation, a new transesophageal echocardiography showed no leak. There were no hemodynamic intercurrences during the procedure. The patient showed clinical improvement of symptoms at first and then he was hemodynamically stable. After a one-year follow-up, the patient progressed with no new hospitalizations due to heart failure, with an improved functional class and 11 mg/dL hemoglobin.

## DISCUSSION

PVL is a serious complication after surgical valve replacement or transcatheter aortic valve replacement, but the incidence of symptomatic patients is low. For years, surgical re-intervention has been considered as the treatment of choice for symptomatic patients with PVL. Transcatheter PVL closure has emerged as a safe, effective, and less invasive alternative to surgical reintervention^[3]^. It requires multiple heart imaging tests and a collaborative team of trained sonographers, anesthetists, hemodynamic technicians and nurses.

In this case, the patient had clear clinical signs of refractory heart failure, hemolytic anemia, and a high EuroSCORE as a result of the previously-cited medical history, with an aggravated clinical condition leading to increased surgical risk; therefore, percutaneous closure was performed. In our experience, although the procedure was technically feasible and successful, there are often potential complications when implanting a device.

The pre-occlusion and post-occlusion follow-up method via transesophageal echocardiography was essential and sufficient for a successful procedure in our experience, but application and development of current heart imaging techniques - such as 3D real-time transesophageal echocardiography and, in particular, angiotomography combined with volume injection and 3D/4D reconstruction - would allow a better study of dehiscence and also facilitate monitoring the intervention, especially the tomography, in situations where a clear image of anatomical spaces and relationships is required, for example, in a transseptal approach of complexly located leaks.

Significantly improved functional class and hemoglobin levels were observed over the patient's clinical course. It is important to note that all devices used nowadays to close these defects have not been designed for this purpose (offlabel indication), and therefore, new devices for PVL treatment are being developed. Amplatzer Vascular Plugs II and III seem to be the best options due to their remarkable serial results. There have been reports on the use of Amplatzer Vascular Plug III with positive outcomes^[4-6]^.

## CONCLUSION

Percutaneous closure of a PVL using Amplatzer Vascular Plug III device implantation in a patient with refractory heart failure, hemolytic anaemia and a high EuroSCORE is a less invasive option than surgical re-intervention, with lower procedural morbidity and mortality.

**Table:** 

Authors' roles & responsibilities	
GEPB	Agreement to be accountable for all aspects of the work in ensuring that questions related to the accuracy or integrity of any part of the work are appropriately investigated and resolved; final approval of the version to be published
GH	Agreement to be accountable for all aspects of the work in ensuring that questions related to the accuracy or integrity of any part of the work are appropriately investigated and resolved; final approval of the version to be published
JA	Agreement to be accountable for all aspects of the work in ensuring that questions related to the accuracy or integrity of any part of the work are appropriately investigated and resolved; final approval of the version to be published
JF	Agreement to be accountable for all aspects of the work in ensuring that questions related to the accuracy or integrity of any part of the work are appropriately investigated and resolved; final approval of the version to be published
RP	Agreement to be accountable for all aspects of the work in ensuring that questions related to the accuracy or integrity of any part of the work are appropriately investigated and resolved; final approval of the version to be published.
